# Study of Phenolic Compounds and Antioxidant Capacity of Spanish Almonds

**DOI:** 10.3390/foods10102334

**Published:** 2021-09-30

**Authors:** Blanca Moreno Gracia, Diego Laya Reig, María José Rubio-Cabetas, María Ángeles Sanz García

**Affiliations:** 1 Área de Laboratorios de Análisis y Asistencia Tecnológica, Centro de Investigación y Tecnología Agroalimentaria de Aragón (CITA), Avda. Montañana 930, 50059 Zaragoza, Spain; bmorenogr@aragon.es (B.M.G.); masanzg@aragon.es (M.Á.S.G.); 2 Centro de Transferencia Agroalimentaria (CTA), Unidad de Cultivos Leñosos, Gobierno de Aragón, Avda. Montañana 930, 50059 Zaragoza, Spain; dlaya@aragon.es; 3 Instituto Agroalimentario de Aragón—IA2, CITA-Universidad de Zaragoza, Calle Miguel Servet 177, 50013 Zaragoza, Spain; 4 Centro de Investigación y Tecnología Agroalimentaria de Aragón (CITA), Unidad de Hortofruticultura, Avda. Montañana 930, 50059 Zaragoza, Spain

**Keywords:** almond, polyphenols, flavonoids, proanthocyanidins, antioxidant capacity, quality, high performance liquid chromatography

## Abstract

Phenolic compounds have an important influence on fruit and nut quality. Almonds have been shown to be rich sources of phenolic compounds, which possess health-beneficial properties. The objectives of the study were to optimize an extraction method to determine the total amount of polyphenols, flavonoids and proanthocyanidins as well as the antioxidant capacity. In addition, the same extract was used for the identification and quantification of flavonoids by HPLC. The study was conducted on 11 Spanish almond genotypes. The results highlight the differences in the content of antioxidants, which add value to the quality of the fruit. It has been shown that genotype may strongly influence antioxidant capacity and total phenolic compounds. In this work, the almonds with higher results were (Belona, Guara and Vialfas) varieties.

## 1. Introduction

Almonds (*Prunus dulcis* (Mill.) D.A. Webb) are considered a nutritious food having interesting bioactive components [1]. Due to their composition, almonds, as part of a healthy diet, provide benefits that can help to address important public health problems such as diabetes [2], hypertension [3] and overall cardiovascular health [4]. Almonds have an average length of 2.3 cm, 1.4 cm width, and 0.8–1.0 cm thickness. The almonds have an external husk that protects them from the environment. The seeds are oval and flattened, sharpened at one end, and rounded at the other. The almond kernel is surrounded by a brown skin, rough with quite noticeable streaks called tegument [5]. As a food, almonds are consumed raw, fried and roasted. They have been demonstrated to be rich sources of phenolic compounds; their incorporation into the human diet is highly recommended [6] and their benefits depend on regular intake and bioavailability [7].

Therefore, crops in Spain have seen an increase in both production quantity and quality. Almond production, suited to a Mediterranean climate, is concentrated in four main areas of the world: the Mediterranean area, western USA (California), central Asia and Australia [8]. According to ALMENDRAVE [9], production in recent years has been increasing (see Table 1). There are many factors that cause a great variability in the content of antioxidants in almonds. It has been demonstrated that the presence or absence of certain nutrients in the soil and the excess or deficit of irrigation can affect the phytochemical composition of almonds [10]. According to Tapia et al. [11], “many factors highly influence the content of phytochemicals in the nuts: genetics of cultivar, harvest season, geographical origin, environmental conditions (temperatures, rainfall and light), soil composition, maturity level, methods of cultivation, processing and storage”.

As a result, it is important to increase our knowledge about almond varieties’ nutraceutical composition. Polyphenols, mainly tannins and flavonoids, are causally linked to almond quality [12]. Flavonoids are a type of phenolic compounds usually found as O-glucosides in almonds [13]. Proanthocyanidins are flavan-3-ol monomers, oligomers and polymers that form anthocyanidins upon hydrolysis in mineral acids [14]. Almond proanthocyanidins mainly consist of (+)-catechin and (−)-epicatechin [15].

**Figure 1:**
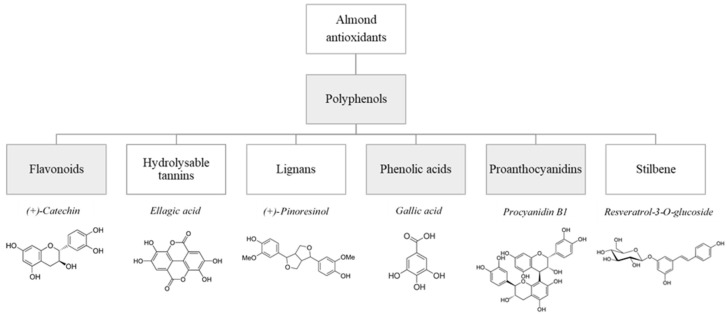
Examples of antioxidants present in almonds.

Figure 1 shows the main antioxidants present in almonds, highlighting the studied compounds.

Almond research started in the present-day CITA de Aragón in 1964. The objectives of the almond breeding program were with three main objectives: self-compatibility to overcome the pollination problems, late blooming in order to have cultivars blooming once the main frost risks were over and finally fruit quality.

One of the first cultivars released was (Guara), which nowadays is the reference cultivar in all the Spanish almond growing regions. The breeding program has been carried on, releasing new cultivars in order to satisfy the quality requirements of the international market with compositional traits more adapted to the commercial and industrial determining factors, such as (Belona) and (Soleta), as well as very late blooming cultivars, such as (Diamar) and (Vialfas). All of them are self-compatible, without the need of cross pollination nor pollinating insects, and with a progressive ripening time allowing a gradual harvest.

Our work is part of Research Project: An Integrative Approach for Optimization of Almond Rootstocks and Scion Breeding. The aim of this study was to quantify the total phenols, flavonoids and proanthocyanidins to determine the main flavonoids present in 11 genotypes of Spanish almonds as a measure of the antioxidant activity.

## 2. Materials and Methods

### 2.1. Chemicals

The following were used in the study: Folin-Ciocalteu’s phenol reagent (Panreac), sodium carbonate, sodium hydroxide, ammonium acetate (Carlo Erba), sodium nitrite 98%, iron (II) sulfate heptahydrate, iron (III) chloride hexahydrate and 2,4,6-tri-(2-pyridyl)-1,3,5-triazine (TPTZ) 98%, (Alpha Aesar), anhydrous aluminum chloride, hydrochloric acid, n-butanol (Merck), Trolox 97% (Acros Organics), gallic acid, (+)-catechin, daidzein (Sigma Aldrich), cyanidine chloride, (−)-epicatechin, kaempferol-3-rutinoside, isorhamnetin-3-*O*-rutinoside, isorhamnetin-3-*O*-glucoside (Extrasynthese). All materials used had a high purity, above 95%.

### 2.2. Almond Samples

Eleven almond cultivars of different origins were used in this study. Five were experimental selections (G-2-22, G-3-3, G-3-4, G-5-25 and I-3-67), and six were commercial cultivars (Belona, Guara, Mardia, Soleta, Vairo and Vialfas). All, except (Vairo), were obtained at the Agrifood Research and Technology Centre (CITA), Aragon (Spain). The genotypes were grown in a 15-year-long trial in AFRUCCAS, (Association of Fruit Growers of the Region of Caspe), an experimental farm in association with the Government of Aragón, located in Caspe (41.315570, 0.085238). All the almond trees were grown under the same soil, irrigation and fertilizer conditions, enabling the comparison of results in this study.

The work was done in four late-blooming genotypes (Belona, Guara, Soleta and Vairo) and two extra late-blooming genotypes (Mardia and Vialfas). The almonds were harvested in their ripening period during August and September 2017. All of them were hard-shell cultivars, with shelling percentage of 27–35% in (Belona) and (Soleta), 24–25% in (Mardia) and (Vialfas), 29% in (Vairo) and 35–40% in (Guara). The kernel of (Belona) was large and rounded, whereas (Soleta) was large and elliptical. The varieties (Guara, Mardia, Vairo and Vialfas) were medium-sized and heart-shaped.

All almonds were equally air-dried. When the shell was fully dried, it was separated, and the whole kernel was stored in vacuum bags. The samples were stored at −20 °C until further analysis. For the analysis, kernels with their tegument were ground in a mortar under liquid nitrogen until completely ground. All samples were done in triplicate. The results were reported as grams per 100 g of wet sample.

### 2.3. Antioxidants Extraction

Antioxidants were extracted using 30 mL of a hydrochloric acid, water and methanol (3.7:46.3:50, *v*/*v*/*v*) solution added to 0.5 g of the ground sample. The solution was stirred using a Muti-Reax at 1700 rpm in the dark for 2 h. Later, the solution was sonicated for 10 min and maintained at 4 °C by adding ice. Finally, the solution was centrifuged (5 min, 3000× *g*, 4 °C). The supernatant was filtered through Whatmann No. 1 filter paper. This process was repeated two more times, with 30 mL stirred for 1 h, and then 20 mL stirred for 30 min. The combined filtrate from three extractions was completed to 100 mL.

### 2.4. Total Polyphenol Determination

Total polyphenols were determined using spectrophotometric techniques with the modified Folin Ciocalteu method by Singleton, Orthofer and Lamuela-Raventós [16]. Briefly, 200 µL of extract was diluted to 1 mL with Milli-Q water and then mixed with 5 mL of Folin-Ciocalteu reagent 1:10 and 4 mL of sodium carbonate 1N. After 1 h, the mix was measured using a spectrophotometer (Shimadzu, UV-1700) at 760 nm. The results were expressed as gallic acid equivalent (GAE).

### 2.5. Total Flavonoid Determination

The method used was as described by Zhishen, Mengcheng and Jianming [17] and modified by Jahanban-Esfahlan and Jamei [18]. It is based on the reaction of aluminum ions with the flavonoid molecules under basic conditions.

The test was carried out with a slight modification. A total of 1.5 mL of the extract was added to 450 µL of 5.3% NaNO_2_, 900 µL of 10% AlCl_3_-H_2_O and 4 mL of 1 M NaOH. The mixture was stirred and let rest for 5 min before each addition. The final volume was completed to 15 mL with Milli-Q water. The absorbance was measured at 510 nm. The results were expressed as (+)-catechin equivalent (CAT).

### 2.6. Total Proanthocyanidin Determination

The method of Ribéreau-Gayon and Stonestreet [19] was followed. Briefly, 1 mL of the extract and 10 mL of 0.54 mM FeSO_4_·7H_2_O in n-butanol/hydrochloric acid (50:50, *v*/*v*) were heated to 90 °C for 1 h. Absorbance was measured at 550 nm. The results were expressed as cyanidin chloride equivalent (CYA).

### 2.7. FRAP Assay

The antioxidant capacity was measured according to Benzie and Strain [20]. A volume of FRAP reagent, prepared with 83.3% of 300 mM ammonium acetate (pH = 3.6) and 16.7% of the mixture (50:50) of tripyridyltriazine (TPTZ) reagent in 40 mM HCl and 20 mM FeCl_3_·6H_2_O was heated to 37 °C for 30 min. The absorbance at 593 nm was recorded as the initial data. Then, 1 mL of the antioxidant extract was added, and once the kinetic reaction was completed, the final absorbance was measured. The results were expressed as µmoles of iron sulfate heptahydrate (Fe^2+^) per 100 g of wet sample.

### 2.8. Determination of Flavonoid Compounds by HPLC

The identification and quantification of phenolic compounds was carried out by liquid chromatography using the initial extract double concentrated using nitrogen stream. The extract was filtered (Nylon, 0.45 µm, 0.25 mm ø) before injection into the chromatograph. A Series 1100 HPLC with a diode-array detector (DAD) and a fluorescence detector (FLD), 1200 Series (Agilent Technologies) was used. The mobile phase was prepared with Milli-Q water acidified with formic acid 1% (A = water phase) and methanol (B = organic phase). Compounds were separated in the following gradient, expressed as a percentage of A: 0–1 min until 95%, 1–25 min until 80%, maintain for 5 min, 30–90 min until 0%, maintain for 10 min. Between the two consecutive injections, a period of 40 min was necessary to equilibrate the column. The column used was an Eclipse XDB-C18 column (150 × 4.6 mm, 4 μm) at a constant flow rate of 1 mL/min and an injection volume of 100 μL. For the identification and quantification of flavonoids, DAD signals were recorded at wavelengths 280 and 360 nm and FLD at 230 and 310 nm, for excitation and emission, respectively. The internal standard was daidzein, an isoflavone found in plant foods [21]. The results were expressed as mg phenolic compound per 100 g of fresh weight.

### 2.9. Method Validation

The applicability of the method for the quantitative determination of antioxidants in samples was demonstrated by performing analytical validation of parameters such as selectivity, linearity, limits of detection (LOD) and quantification (LOQ), recovery and precision, calculated as the repeatability and reproducibility, following the criteria of the Eurachem Guide [22]. Reagent blanks, with and without standard addition, as well as fortified samples at different levels were studied for selectivity. The linearity of the method was determined by performing in triplicate, on three different days, the external calibration curve obtained from standard solutions and calculating the regression line by the method of least squares and the coefficients of determination, R^2^. In addition, a study of the slopes was carried out, and with these the linearity coefficients were obtained.

The detection and quantification limits for spectrophotometric determinations were calculated as 3 and 10 times the average standard deviation of 10 target determinations divided by the slope of the standard curves. In chromatography, they were determined as the ratio of the lowest concentration of the calibration line to the signal/noise expressed in concentration, applying a factor of three and six times for the limits of detection and quantification, respectively. The injection precision was calculated with five consecutive injections of the same sample. The recovery (expressed in %) was calculated from fortified samples at different levels, which were established considering the range of the calibration curve of the method, the limit of quantification and the midpoint of the calibration curve. Five repetitions of each spiking level were performed, and 10 were unfortified.

The precision, expressed as relative standard deviation (%), was established in terms of repeatability and reproducibility, and for this purpose five repetitions were performed under the same conditions, repeated on three independent days with different analysts.

### 2.10. Statistical Analysis

All statistical analyses in this work were performed using the XLSTAT statistical package (ver. 2020.4.1.1015) from Microsoft Excel, and the results are shown as an average ± standard error. Analysis of variance (ANOVA) was performed, followed by Tukey’s test (*p* ≤ 0.05), to establish significant differences between means. For correlation, Pearson’s linear coefficient was used. Finally, principal component analysis (PCA) was applied to visualize differences and similarities between study genotypes.

## 3. Results and Discussion

### 3.1. Antioxidant Extraction

Many parameters can influence the antioxidant recovery from almond shells, such as the liquid–solid ratio for each solvent, the temperature and time of extraction [23], as well as the polarity of the solution and sample concentration [24]. Sample size and stirring can also have an effect on extraction. The parameters studied in this work for extraction process optimization of whole almonds (kernel and tegument) were as follows: solvent, sample ratio, solvent mixture and total volume, stirring conditions and number of extractions.

Two different polar solutions were selected for this work: MeOH:HCl (1000:1, *v*/*v*) and HCl:H_2_O:MeOH (3.7:46.3:50, *v*/*v*/*v*). The first dissolution was used to extract fractions rich in antioxidant compounds from industrial processing of almond by-products, including the skin, shell and mesocarp [25]. The second dissolution was used to identify and quantify 20 phenolic compounds by high-performance liquid chromatography [26]. With the latter method, higher values of total polyphenols were obtained, and more phenolic compounds were identified chromatographically in our samples; thus, this dissolution was chosen for further analysis. Three different sample amounts were tested: 0.5, 1 and 3 g. A weight of 0.5 g was selected because larger amounts of sample require higher volumes of solvent to achieve total extraction of antioxidant compounds and make its chromatographic detection difficult.

The use of a Multi-Reax^®^ instrument (Heidolph Instruments GmbH & CO, Schwabach, Germany), with shaking at room temperature and controlled speed, was contrasted with ultrasound at temperatures not exceeding 4 °C. In addition, different stirring times were tested (1, 2 and 3 h). The combination of both types of stirring during 3.5 h was finally selected for a more efficient extraction. The positive effect of ultrasound-assisted extraction on the results has been previously cited [27,28]. With microwave irradiation, a higher content of extractable bioactive compounds was obtained, justified by the effects of acoustic cavitation and subsequent internal heating in plant cells, producing an increase in structural damage and thus improving the release of the compounds [29].

The extraction temperature was 25 °C. Temperature is the most significant parameter in antioxidant extraction because at higher temperatures some phenolic compounds can be destroyed [30]. The final extraction volumes were 10, 30, 50, 100 and 200 mL, and it was concluded that the best results were obtained with a final volume of 100 mL. To verify the total extraction of antioxidant compounds, the sample residue was analyzed. Residual percentages of 1.4% and 1.3% of the total polyphenols and flavonoids, respectively, were obtained, so the extraction of antioxidants with the proposed method was considered optimal.

### 3.2. Validation of Analytical Methods

Table 2 shows the data obtained for the different validation parameters studied using the spectrophotometric methods.

The representation of the concentration (mg/L) against the signal showed a linear ratio in all cases that could be adjusted by least squares with values of correlation coefficients (R^2^) higher than 0.99 and linearity coefficients higher than 93%. The limits of detection and quantification obtained in the content of total compounds varied between 0.3 and 1 µg/mL for LOD and between 1.0 and 3 µg/mL for LOQ, with the lowest values corresponding to proanthocyanidins. Similar values were obtained for total polyphenols and flavonoids, with limits of detection of 1.8 and 0.9 µg/mL and limits of quantification of 5.4 and 2.7 µg/mL, respectively [31]. Dini et al. [32] published, for olive oil, limits of detection and quantification in total polyphenols of 1.0 and 2.0 µg/mL, respectively.

In the precision study, both in terms of repeatability and reproducibility, the largest relative deviations in the determination of total polyphenols were obtained. The recovery studies were performed with samples that were spiked with different amounts of external standards: gallic acid, (+)-catechin, and cyanidine chloride. Our results, expressed as percentage recovery, range from 88 to 113%, as shown in Table 3; similar results for both parameters have previously been published [31,33]. Values obtained in FRAP assay in whole almonds were similar to values in studies on the tegument of seven American varieties [21]. The recovery of the antioxidant capacity in our FRAP assay had values of 107 and 120%, depending on the level of concentration. Ferrous sulfate heptahydrate was used as an external standard.

Table 4 presents the results of the validation parameters of the flavonoids analyzed by chromatography. In the five flavonoids studied, the linearity was corrected with coefficients of determination above 0.980 and injection accuracy below 4%. The detection and quantification limits obtained were lower than those published in other studies [28,33].

Recovery was calculated by fortifying samples with 0.12 µg/mL for flavan-3-ol and 0.20 µg/mL for flavonols, obtaining recoveries between 96% and 112%, with a lower value of 82% corresponding to catechin recovery.

### 3.3. Analysis of Almond Genotypes

The results for total compounds of the 11 Spanish almond genotypes are presented in Table 5. The statistical analysis showed significant differences related to genotype.

In the case of total polyphenols, these differences divide the samples into five groups, flavonoids into six and proanthocyanidins into four. The results for total polyphenols showed the highest values in genotypes (Guara and Vialfas), being 1.5–2 times higher than other genotypes. Belona and Vialfas had a higher content of flavonoids and total proanthocyanidins, followed by (Guara and G-3-4). In addition, antioxidant capacity was higher in the previous genotypes, in agreement with the values of the total determinations. In all cases, the (G-2-22) genotype presented the greatest significant differences.

The mean value of polyphenols in the samples analyzed was 373.1 mg GAE/100 g, which is higher than that published in the review of nuts, with a total polyphenol value in almonds of 261 mg GAE/100 g [34]. In the varieties (Father) and (Price), respective average values of 199.5 and 240.8 mg GAE/100 g have been published [26]. Greater variability was obtained in the studies of 10 varieties of wild almonds from Iran, with ranges of total polyphenol values between 184.1 and 482.3 mg GAE/100 g, and in eight varieties of Portuguese almonds, with results between 32 and 347 mg GAE/100 g, corresponding to the (Bonita) and (Casanova) varieties, respectively [18,35]. In detail, the cultivars examined in the study [36] were the Australian (Johnston Prolific), the Californian (Texas) and (Thompson), the Italian (Filippo Ceo, Genco and Tuono), the Spanish (Desmajo Largueta) and (Marcona), as well as (Francolì) and (Ferragnès). The total phenolic compounds showed a great variability among cultivars, ranging from 39.20 (Marcona) to 1103.05 mg/100 g GAE (Genco) on dry matter. Similar to our findings were the results of the varieties (Ferragnès) and (Tuono) with (Belona) and (Guara).

The average total flavonoid content value in the analyzed almonds was 132.4 mg CAT/100 g. Flavonoid data published in the literature for samples of almonds are lower than those obtained in this study; the extraction process may be the main source of this difference. In eight varieties of American almonds, published values are between 14.56 and 27.18 mg CAT/100 g [26]. The total flavonoid content in the (Guara) variety was quantified in the range of 6.24–25.02 mg CAT/100 g as a function of the extraction mixture used [30]. Different reviews have published a mean value of total flavonoids in almonds of 25.01 mg CAT/100 g and a range of 13.0–93.8 mg CAT/100 g [34,37].These values are in the same order as the data obtained in the work of Jahanban-Esfahlan and Jamei [18], from 11.3 to 35.6 mg CAT/100 g. Yildiz et al. [38], in their study of 24 genotypes of almonds harvested in Turkey, obtained a range between 15.11 and 51.15 mg CAT/100 g for the varieties (Picantalli) and (Garrigues), respectively.

The content of total proanthocyanidins (Table 5) has been published in few studies. In the 11 genotypes studied, the average value was 196.6 mg CYA/100 g, in line with that published in review papers, which quote a value of 184.1 mg/100 g [34]. In American varieties, 110.6 mg CYA/100 g was obtained for the (Nonpareil) variety, and lower values were found for the (Butte and Carmel) varieties [12]. In general, the variation in published proanthocyanidin content is substantial, with values between 67.1 and 257 mg CYA/100 g [37].

There are many factors that can cause great variability in the antioxidant content in almonds [39]. Among the extrinsic factors are growing conditions and storage after harvest. It has been shown that the presence or absence of certain nutrients in the soil and the excess or deficit of irrigation can affect the phytochemical composition of fruits and vegetables [10]. The degree of maturity, illumination and irradiation of the plants, together with the temperature, also has a great influence on the content of phytochemicals [40].

Data on antioxidant capacity obtained by the FRAP test are shown in Table 5. The highest values of antioxidant capacity corresponded to the varieties (Vialfas, Guara and Belona), which also showed the highest content of polyphenols, flavonoids and total proanthocyanidins. Significant differences divide the 11 samples into five groups, two in the range of 4500–6000 μmol Fe^2+^/100 g and three between 7900 and 9800 μmol Fe^2+^/100 g. The highest antioxidant capacity values, seen in the (Nikitski) (6169 μmol Fe^2+^/100 g) and (Garrignes) (6146 μmol Fe^2+^/100 g) varieties, and the lowest result, seen in the (Gulcan) variety (2088 μmol Fe^2+^/100 g), have been previously reported [38]. However, a previous study reported that, in the tegument of the almond variety (Avola), the average value was 6034.5 μmol Fe^2+^/100 g [41].

The statistical correlation analysis (Pearson) of data obtained from the FRAP assay and the total compound determinations showed a high correlation. The highest correlations were obtained between the FRAP assay and total proanthocyanidins (0.976) and total polyphenols (0.944). Other authors found similar correlations between the FRAP assay and polyphenols: 0.961 [18], 0.926 [42], 0.916 [36] and 0.997 [41]. For the correlation of the antioxidant capacity and total flavonoids, the value obtained in this work was 0.932, which is higher than was cited (0.893) in the work of Jahanban-Esfahlan and Jamei [18]. In our study, the lowest correlation was obtained between polyphenols and total flavonoids (0.844).

**Figure 2:**
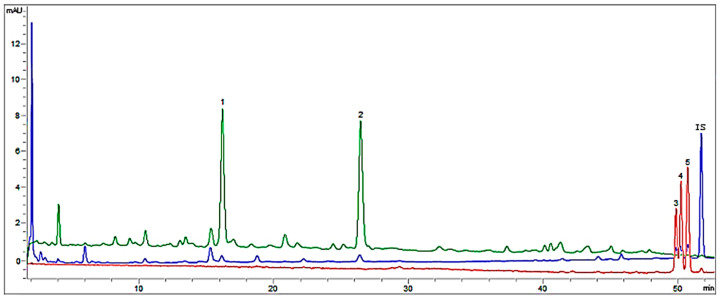
Chromatogram of a sample with added internal standard. Blue signal DAD = 280 nm, red signal DAD = 360 nm, green signal FLD Ex = 230 nm and Em = 280 nm. Peaks identified and quantified by HPLC 1: (+)-catechin; 2: (−)-epicatechin; 3: isorhamnetin-3-*O*-glucoside; 4: kaempferol-3-*O*-rutinoside; 5: isorhamnetin-3-*O*-rutinoside; IS: daidzein.

In the high performance liquid chromatography analysis, five polyphenolic compounds corresponding to two different flavonoid subclasses were identified: two flavon-3-ols—(+)-catechin and (−)-epicatechin; three flavonols—isorhamnetine-3-*O*-rutinoside, isorhamnetine-3-*O*-glucoside and kaempferol-3-*O*-rutinoside. These last two flavonol glycosides have also been identified in American almonds [43]. Figure 2 shows the chromatogram of a sample with the peaks identified, and Table 6 shows the quantified flavonoid results. Catechin was the majority compound, except for the (I-3-67) and (Belona) varieties; in all cases, values were greater than those previously published [6,37].

The sum of flavan-3-ols represents 62–85% of the total detected flavonoids with values between 14.14 and 29.93 mg/100 g fw. Barral-Martinez et al. [44] assembled lower results in whole almonds of flavan-3-ols (1.27–5.13 mg/100 g fw) compared to those of our study (14.33–29.93 mg/100 g fw). However, they obtained higher results of flavanols (11.3–23.1 mg/100 g fw). 

The eight American varieties (Butte, Carmel, Fritz, Mission, Monterey, Nonpareil, Padre, and Price) [26] were provided in raw, whole form showing lower values of (+)-catechin (0.95–3.86 mg/100 g fw) and (−)-epicatechin (0.32–1.27 mg/100 g fw) than those obtained in our study.

Similar to our findings were the results of almond kernels from Turkey [45] in phenolic compounds (+)-catechin and (-)-epicatechin. However, in our work other flavanols were quantified. The kaempferol-3-*O*-rutinoside flavonol showed the lowest concentrations, in accordance with Chang et al. [6] and the review of Bolling [37]. In addition, they assembled mean values of (+)-catechin (1.46 and 5.40 mg/100 g fw) and (-)-epicatechin (0.85 and 2.11 mg/100 g fw) in whole almonds. All the Spanish almond genotypes showed higher results in the content of these flavan-3-ols, highlighting (Belona) and (Guara) varieties. These authors [6,37] found studies with higher results of flavanols—isorhamnetine-3-O-rutinoside, isorhamnetine-3-O-glucoside and kaempferol-3-O-rutinoside. In 12 Serbian regional almonds cultivars [46], the content of (+)-catechin (1.52–11.60 mg/100 g fw) was studied, showing the variety (Tuono) the highest result. Other distinct flavonoids—naringenin-7-*O*-glucoside, dihydrokaempferol and naringenin—have previously been studied by analyzing almond tegument [21]. The sum of the five detected compounds in the genotypes studied was between 14% and 30% of the total flavonoids; Chang et al. [6] found 6–11% in the tegument of different varieties of almonds.

The content of individual flavonoids could be used as discriminating parameters to differentiate almond varieties since they seem to depend more on the almond cultivar than on other variables [5].

**Figure 3:**
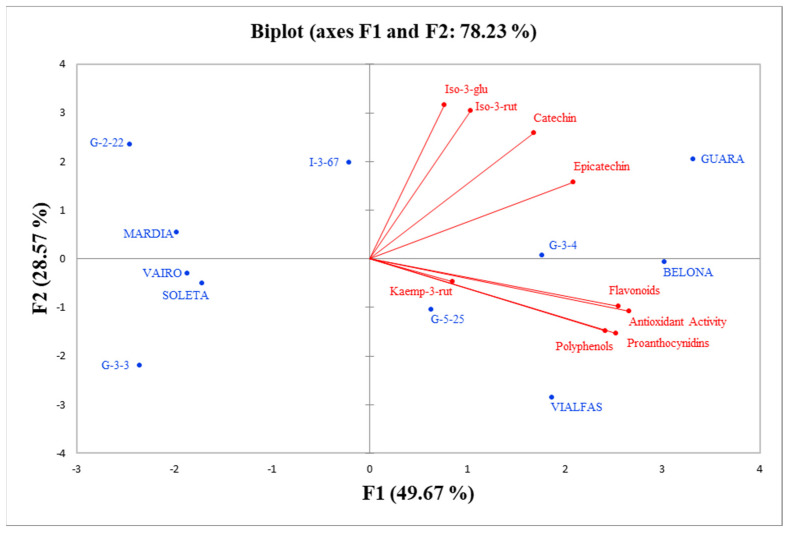
Biplot of the first two components of the PCA.

The principal component analysis (PCA) is shown in Figure 3. It was performed by configuring a matrix consisting of nine variables and 11 genotypes. The first two components, F1 and F2, explain 78.23% of the variability, a percentage sufficiently high to ensure that the PCA plots were representative of the main features of the data set.

The F1 component explains 49.67% of the variability in the data. The variables’ *p*-values and contributions (%) are as follows: antioxidant activity (0.944, 19.94%), total flavonoids (0.907, 1.41%), total proanthocyanidins (0.898, 18.02%) and total polyphenols (0.859, 16.52%). The F2 component explains 28.56% of the variability, and the variables with the highest *p*-value and contribution (%) are as follows: isorhamentin-3-*O*-glucoside (0.852, 28.27%), isorhamentin-3-*O*-rutinoside (0.822, 26.30%), catechin (0.696, 18.84%) and epicatechin (0.426, 7.06%). The former two variables showed a strong correlation with each other (0.719) and slightly lower with the latter two variables. The first component, F1, which corresponds to a gradient in antioxidant activity, flavonoids, proanthocyanins and total polyphenols, allows for clear separation into two groups of genotypes: the first with high values of all variables (G-3-4, G-5-25, Belona, Guara and Vialfas) and the second with low values (G-2-22, G-3-3, Diamar, Vairo and Soleta), leaving only genotype (I-3-67) in an intermediate position. In contrast, the second component, F2, corresponds to a gradient in isorhamentine-3-*O*-glucoside, isorhamentine-3-*O*-rutinoside and catechin that does not separate genotypes into clearly contrasting groups. In this gradient (G-2-22, I-3-67 and Guara) show maximum values and (Vialfas and G-3-3) show minimum values.

## 4. Conclusions

In this work, an extraction method was optimized to analyze total antioxidants in almonds, including phenols, flavonoids and proanthocyanidins. The same extract can be applied to the separation and quantification of flavonoids by HPLC and, in addition, to the study of antioxidant capacity. Control of all the parameters affecting the polyphenol extraction process is fundamental; the validation showed that it is appropriate for almond composition determinations. The methods were applied for different genotypes of Spanish almonds, and it was found that the composition of compounds is different between varieties. The statistical analysis showed that the 11 analyzed genotypes can be easily separated into two groups. One group, with genotypes (G-3-4, G-5-25, Belona, Guara and Vialfas), is strongly correlated with antioxidant activity and total steady compounds. The second group comprises genotypes that are not correlated: (G-2-22, G-3-3, Mardia, Soleta and Vairo). The variety (Belona) had 65.40% of oil and 75.60% of oleic acid in its composition, as well as antioxidants, mainly tocopherols and compound phenolics, thus resulting in one of the cultivars of extremely high quality. A large number of phenolic compounds were obtained in the (Guara) almonds, highlighting their high content of flavan-3-ols(+)-catechin and (−)-epicatechin 16.58 and 13.35 mg/100 g fw, respectively. In (Vialfas) the content of oleic acid was extremely high (77.97%), as well as the amount of total polyphenols were one of the highest in our study—476.4 mg GAE/100 g. In addition, these varieties showed the highest antioxidant capacity. All main traits provide quality to the fruit. The three genotypes described would be the most recommended for consumers from a nutritional and quality point of view.

Whole almonds were used to assess nutritional value in this study; however, analysis of the tegument alone may be of interest in further research, as it may allow for an increased number of flavonoids to be identified.

## Figures and Tables

**Table 1:** Almond production estimation for 2019/2020 campaign in Spain.

Autonomous Community	Production 2018 (Almond Kernel, t)	Forecast 2019 (Almond Kernel, t)	Variation 2018–2019	Variation 2019 over Average 2014–2018
Andalucia	11,500	14,950	+30.00%	+28.15%
Aragon	18,588	16,850	−9.35%	+7.78%
Baleares	1250	1000	−20.00%	−24.03%
Castilla La Mancha	11,666	7954	−31.82%	+9.08%
Cataluña	4563	6533	+43.17%	+60.76%
La Rioja	250	400	+60.00%	−36.16%
Murcia	5520	5800	+5.07%	+7.87%
Extremadura	2000	2460	+6.00%	+146.00%
Comunidad Valenciana	6500	6890	+23.00%	+22.55%
Other	190	200	+5.26%	−5.70%

Note. Adapted from Production estimation for 2019/2020 campaign in Spain, by Group of Almond and Hazelnut Exporters of Spain, 2019 (https://www.almendrave.com/el-sector/produccion, accessed on 15 March 2021) [9].

**Table 2:** Validation parameters for antioxidant determination by spectrophotometry.

	Linear Equation	R^2^	Coefficient Linearity (%)	LOD (μg/mL)	LOQ (μg/mL)	Repeatability (%RSD)	Reproducibility (%RSD)
Total polyphenols	0.0022x + 0.0036	0.9949	99	1.0	3.0	8.5	9.0
Total flavonoids	0.0036x + 0.0018	0.9987	96	2.0	2.0	5.5	6.5
Total proanthocyanidins	0.0058x + 0.0015	0.9965	93	1.0	1.0	6.8	7.3
FRAP assay	0.0069x − 0.00036	0.9997	98	1.0	1.0	2.8	3.2

**Table 3:** Antioxidant recovery determinations by spectrophotometry.

	Added Concentration (μg/mL)	Recovery (%)
Total polyphenols	25	99
	75	96
	100	104
Total flavonoids	8	88
	15	113
Total proanthocyanidins	2	107
FRAP assay	25	120
	50	107

**Table 4:** Validation parameters in HPLC flavonoid determination.

	Linear Equation	R^2^	Coefficient Linearity (%)	LOD (μg/mL)	LOQ (μg/mL)	Accuracy Injection (%RSD)	Repeatability (%RSD)	Reproducibility (%RSD)
(+)-Catechin	108.7x − 3.6	0.9937	99	0.01	0.02	4.0	6.4	9.3
(−)-Epicatechin	107.1x − 4.0	0.9848	99	0.02	0.03	0.6	4.7	10.6
Isorhamnetin-3-*O*-glucoside	159.4x + 0.59	0.9988	99	0.01	0.03	1.3	1.9	6.5
Kaempferol-3-*O*-glucoside	219.4x − 3.3	0.9808	100	0.007	0.01	1.8	3.3	6.6
Isorhamnetin-3-*O*-rutinoside	175.7x + 0.92	0.9961	99	0.01	0.02	0.3	2.0	10.9

**Table 5:** Total results of polyphenols, flavonoids, proanthocyanidins and antioxidant capacity in the samples.

Genotype	Polyphenols (mg GAE/100 g)	Flavonoids (mg CAT/100 g)	Proanthocyanidins (mg CYN/100 g)	FRAP Assay (µmol Fe^2+^/100 g)
G-2-22	245.2 ± 8.2 ^e^	105.7 ± 1.6 ^f^	103.4 ± 3.2 ^d^	4507.1 ± 153.9 ^e^
G-3-3	359.9 ± 8.2 ^c^	122.0 ± 2.3 ^cde^	163. 9 ± 1.7 ^c^	5405.3 ± 47.5 ^de^
G-3-4	422.7 ± 16.6 ^b^	149.5 ± 1.5 ^b^	236.1 ± 4.3 ^b^	8256.3 ± 135.7 ^bc^
G-5-25	438.6 ± 21.8 ^ab^	127.2 ± 3.4 ^cd^	216.1 ± 5.1 ^b^	7898.5 ± 337.8 ^c^
I-3-67	299.1 ± 8.7 ^de^	133.3 ± 2.0 ^c^	157.1 ± 6.7 ^c^	5817.5 ± 79.4 ^d^
BELONA	424.9 ± 10.2 ^b^	156.0 ± 2.2 ^ab^	281.3 ± 11.1 ^a^	9077.5 ± 320.1 ^ab^
MARDIA	307.9 ± 12.9 ^cd^	113.3 ± 2.0 ^ef^	154.8 ± 3.9 ^c^	5406.2 ± 100.4 ^de^
GUARA	486.8 ± 4.7 ^a^	151.7 ± 2.2 ^b^	240.3 ± 9.4 ^b^	9137.3 ± 77.6 ^ab^
SOLETA	324.7 ± 6.4 ^cd^	112.3 ± 4.9 ^ef^	153.9 ± 1.3 ^c^	5791.9 ± 187.5 ^d^
VAIRO	317.9 ± 2.4 ^cd^	118.3 ± 2.2 ^def^	169.1 ± 0.8 ^c^	5959.9 ± 82.7 ^d^
VIALFAS	476.4 ± 19.9 ^ab^	168.1 ± 3.3 ^a^	286.6 ± 8.0 ^a^	9785.4 ± 178.1 ^a^

Mean ± standard error (*n* = 3). Different letters show statistical significance (*p* ≤ 0.05) between different samples.

**Table 6:** Phenolic compounds in the samples identified and quantified by HPLC-DAD-FLD.

Genotype	(+)-Catechin	(−)-Epicatechin	Isorhamentin-3-*O*-glucoside	Kaempferol- 3-*O*-rutinoside	Isorhamentin-3-*O*-rutinoside	Sum Flavan-3-ols	Sum Flavanols
G-2-22	10.79 ± 1.57 ^ab^	5.50 ± 0.34 ^c^	2.74 ± 0.21 ^bc^	0.11 ± 0.10 ^c^	8.35 ± 0.37 ^a^	16.29	11.21
G-3-3	9.69 ± 0.79 ^ab^	5.52 ± 0.30 ^c^	0.54 ± 0.05 ^f^	<LOQ	2.01 ± 0.04 ^d^	15.21	2.55
G-3-4	12.82 ± 0.70 ^ab^	9.34 ± 0.68 ^abc^	1.64 ± 0.09 ^de^	0.31 ± 0.04 ^bc^	5.20 ± 1.25 ^bc^	22.16	7.15
G-5-25	8.72 ± 0.32 ^ab^	6.24 ± 0.17 ^c^	0.87 ± 0.04 ^ef^	0.53 ± 0.07 ^b^	3.29 ± 0.17 ^cd^	14.96	4.69
I-3-67	11.57 ± 1.94 ^ab^	11.92 ± 2.02 ^ab^	3.87 ± 0.22 ^a^	<LOQ	7.11 ± 0.28 ^ab^	23.49	10.98
BELONA	12.80 ± 0.54 ^ab^	13.81 ± 1.04 ^a^	2.67 ± 0.14 ^bc^	0.96 ± 0.05 ^a^	5.46 ± 0.55 ^bc^	26.62	9.10
MARDIA	8.89 ± 1.59 ^ab^	5.25 ± 0.51 ^c^	2.29 ± 0.14 ^cd^	0.11 ± 0.09 ^c^	4.14 ± 0.26 ^cd^	14.14	6.54
GUARA	16.58 ± 3.22 ^a^	13.35 ± 1.67 ^a^	3.51 ± 0.03 ^ab^	<LOQ	6.80 ± 0.28 ^ab^	29.93	10.32
SOLETA	8.46 ± 1.00 ^b^	7.10 ± 0.63 ^bc^	1.26 ± 0.09 ^ef^	0.40 ± 0.09 ^b^	3.42 ± 0.11 ^cd^	15.56	5.08
VAIRO	8.21 ± 1.39 ^b^	6.12 ± 0.92 ^c^	1.67 ± 0.36 ^de^	<LOQ	4.29 ± 0.88 ^cd^	14.33	5.96
VIALFAS	9.31 ± 1.50 ^ab^	6.35 ± 0.80 ^c^	0.67 ± 0.07 ^f^	<LOQ	3.53 ± 0.27 ^cd^	15.66	4.20

Mean ± standard error in mg/100 g fw (*n* = 3). Different letters show statistical significance (*p* ≤ 0.05) between different samples.

## Data Availability

Data are contained within the article.
